# Association of Race/Ethnicity With Overall Survival Among Patients With Colorectal Liver Metastasis

**DOI:** 10.1001/jamanetworkopen.2020.16019

**Published:** 2020-09-09

**Authors:** Lucas W. Thornblade, Susanne Warner, Laleh Melstrom, Gagandeep Singh, Yuman Fong, Mustafa Raoof

**Affiliations:** 1 Department of Surgery, City of Hope National Medical Center, Duarte, California

## Abstract

This cohort study assesses the association of race/ethnicity with overall survival among patients with colorectal liver metastasis overall and among those undergoing liver resection.

## Introduction

Black patients in the US experience lower rates of survival after surgery for colorectal cancer (CRC).^1^ Disparity in outcomes after cancer surgery is associated with a complex interaction of patient, socioeconomic, and health-system factors, including lower rates of referral to cancer specialists,^2^ higher risk factors for CRC, and patient reported barriers, including fear of cancer and its treatment, costs, and the burdens of transportation and childcare during therapy.^3^

Once considered inoperable, for many patients, metastatic CRC is now viewed as a curable disease. However, few people with colorectal liver metastases (CRLM) undergo resection (10% in California).^4^ We assessed whether this low rate of liver resection is associated with lower survival among Black patients with CRC.

## Methods

We performed a retrospective cohort study of adult patients in the California Cancer Registry with synchronous CRLM from January 1, 2000, to December 31, 2012. Data were linked to records from the Office of Statewide Health Planning and Development. Race/ethnicity was recorded by institutional trained abstractors. The use of these data was approved by the Institutional Review Board at City of Hope and by the California State Committee for the Protection of Human Subjects. Informed consent was waived because data were deidentified. This study followed the Strengthening the Reporting of Observational Studies in Epidemiology (STROBE) reporting guideline.

Data were analyzed from August 15, 2019, to January 25, 2020. Adjusted Cox proportional hazard analysis was applied to assess the risk of death, and 2-sided χ^2^ tests measured differences in categorical variables. *P* *<* .05 was considered statistically significant. Analyses were performed using Stata, version 11 (StataCorp LLC).

## Results

Of 16 382 patients (53% male), Black patients had the lowest median survival (11 months) compared with Asian (14 months), Hispanic (14 months), Middle Eastern (18 months), and White (12 months) patients (Figure). Compared with White and Hispanic patients, Black patients were the least likely to receive chemotherapy (59% vs 65% [White patients] vs 68% [Hispanic patients]; *P* < .001) or undergo liver resection (6.2% vs 10.3% [White patients] vs 9.5% [Hispanic patients]; *P* < .001). After controlling for age, sex, comorbidities, and extrahepatic metastasis, Black patients had a 17% higher hazard of death compared with White patients (hazard ratio, 1.17; 95% CI, 1.10-1.24; *P* < .001). Among patients who underwent liver resection for CRLM, there was no difference in survival between Black and White patients (hazard ratio, 1.01; 95% CI, 0.94-1.08; *P* = .84).

**Figure.**
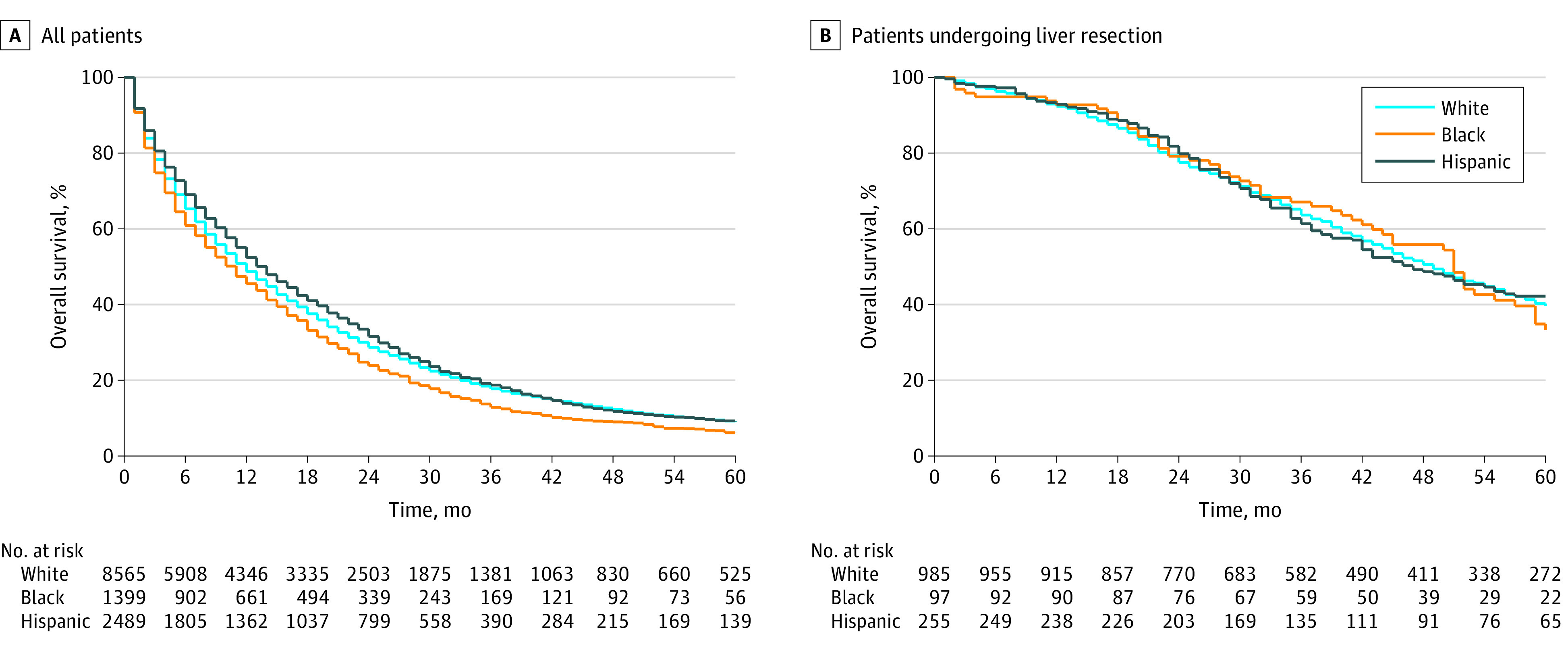
Survival by Racial/Ethnic Group Among Patients With Colorectal Liver Metastasis and Those Who Underwent Liver Resection

## Discussion

We found that Black patients were least likely to undergo chemotherapy or surgical resection for CRLM and had the worst survival compared with patients in other racial/ethnic groups. Benchmarks for improving survival among patients in minority populations who have cancer include maximizing opportunities to visit a cancer specialist, optimizing chances of undergoing state-of-the-art surgical therapy, and ensuring receipt of adjuvant therapy when appropriate.^5^ Although Black race was independently associated with poor survival among patients with CRLM, this study highlights the importance of access to health care systems that perform safe liver resection. The finding that access to surgical resection may be associated with reduced racial differences in survival has been mirrored for other cancers.

Hospital quality may be an important mediator in the association of race/ethnicity and mortality because hospitals account for 54% of excess mortality among Black patients with CRC.^6^ Differences between survival among Black and White patients may in part reflect differences between facilities where patients with stage IV CRC receive chemotherapy vs centers focused on aggressive resection and liver-targeted therapy for select patients. A limitation of this study is that we were unable to adjust for differences in resectability of liver metastases that may exist between racial/ethnic groups.

 These data may provide a basis for a future quality benchmark that all patients with CRLM should be evaluated for resection by a liver surgeon in the office or tumor board setting.
